# Cost-effectiveness of medical primary prevention strategies to reduce absolute risk of cardiovascular disease in Tanzania: a Markov modelling study

**DOI:** 10.1186/s12913-016-1409-3

**Published:** 2016-05-17

**Authors:** Frida N. Ngalesoni, George M. Ruhago, Amani T. Mori, Bjarne Robberstad, Ole F. Norheim

**Affiliations:** Ministry of Health and Social Welfare, Dar es Salaam, Tanzania; Muhimbili University of Health and Allied Sciences, Dar es Salaam, Tanzania; Centre of International Health, University of Bergen, Bergen, Norway; Department of Global Public Health and Primary Health Care, University of Bergen, Kalfarveien 31, Post box 7804, NO-5020 Bergen, Norway

**Keywords:** Sub-Saharan Africa, Tanzania, Primary prevention, Cardiovascular disease, Diabetes, Cost-effectiveness analysis, Markov modelling, Societal perspective

## Abstract

**Background:**

Cardiovascular disease (CVD) is a growing cause of mortality and morbidity in Tanzania, but contextualized evidence on cost-effective medical strategies to prevent it is scarce. We aim to perform a cost-effectiveness analysis of medical interventions for primary prevention of CVD using the World Health Organization’s (WHO) absolute risk approach for four risk levels.

**Methods:**

The cost-effectiveness analysis was performed from a societal perspective using two Markov decision models: CVD risk without diabetes and CVD risk with diabetes. Primary provider and patient costs were estimated using the ingredients approach and step-down methodologies. Epidemiological data and efficacy inputs were derived from systematic reviews and meta-analyses. We used disability- adjusted life years (DALYs) averted as the outcome measure. Sensitivity analyses were conducted to evaluate the robustness of the model results.

**Results:**

For CVD low-risk patients without diabetes, medical management is not cost-effective unless willingness to pay (WTP) is higher than US$1327 per DALY averted. For moderate-risk patients, WTP must exceed US$164 per DALY before a combination of angiotensin converting enzyme inhibitor (ACEI) and diuretic (Diu) becomes cost-effective, while for high-risk and very high-risk patients the thresholds are US$349 (ACEI, calcium channel blocker (CCB) and Diu) and US$498 per DALY (ACEI, CCB, Diu and Aspirin (ASA)) respectively. For patients with CVD risk with diabetes, a combination of sulfonylureas (Sulf), ACEI and CCB for low and moderate risk (incremental cost-effectiveness ratio (ICER) US$608 and US$115 per DALY respectively), is the most cost-effective, while adding biguanide (Big) to this combination yielded the most favourable ICERs of US$309 and US$350 per DALY for high and very high risk respectively. For the latter, ASA is also part of the combination.

**Conclusions:**

Medical preventive cardiology is very cost-effective for all risk levels except low CVD risk. Budget impact analyses and distributional concerns should be considered further to assess governments’ ability and to whom these benefits will accrue.

## Background

Cardiovascular disease (CVD) is a growing cause of death and disability in sub-Saharan Africa (SSA). The Global Burden of Disease (GBD) 2010 study showed that, in 2000, ischemic heart disease and stroke accounted for 1.83 and 2.47 % respectively of total disability- adjusted life years (DALYs) lost in the region. A decade later there had been a 15 % increase in the percentage of total DALYs lost attributed to these conditions [1]. This surge has been boosted by ongoing nutritional, demographic and epidemiological transitions. Costs ascribed to CVD are substantial; for example, in 2010, they amounted to about US$11.6 billion in the World Health Organization’s (WHO) African region E (AFRO E). These costs are expected to rise by 22 % by 2030 [2]. Tanzania has not been spared from this tide, as evidence shows that the prevalence of CVD risk factors is increasing rapidly [3–8]. Consequently, large increases in avoidable CVD mortality and morbidity and hence serious pressures on the already constrained health systems are expected in the future unless preventive measures are implemented.

Many studies on the cost-effectiveness of preventive interventions into CVD have been conducted in high-income countries, with significant amounts of evidence in favour of primary prevention [9]. Evidence from individual countries in SSA is extremely scarce [10–12]. Only a few studies focusing on interventions across WHO and World Bank developing regions are available [13–15], and their use of aggregated data from countries with great diversity in terms of demographic, epidemiological, socio-economic and policy contexts requires the results to be interpreted with caution when used to inform policy in individual countries.

Preventive cardiology in Tanzania not only receives low priority but it is also practised in a non-comprehensive way [16–18]. Tanzania’s previous and current preventive guidelines [19, 20] focus unilaterally on single risk factors but this approach has been shown to be less effective than the absolute risk approach advocated in the WHO’s CVD preventive guidelines and elsewhere [10, 16, 21, 22]. The absolute risk approach gives a numerical probability of a CVD event occurring in a given time period, e.g. one or ten years. It combines all modifiable major risk factors for CVD such as hypertension, cholesterol level and smoking. The absolute risk approach also includes unmodifiable factors like age and sex. Treatment decisions are then based on the total risk of a CVD event [23].

There is a previous study that has explored the cost-effectiveness of medical preventive cardiology in Tanzania [12]. Being the first, it forms an important step for further exploration on the subject. A new study is justifiable for the following reasons: firstly, the previous study did not explicitly model diabetes in its risk factor profile. Existing literature indicates that diabetes is one of the major risk factors for CVD [24] and tends to occur together with other known cardiovascular risk factors [25]. Secondly, primary cost data were not used in the estimation of intervention costs. Thirdly, the current work expands from a provider to a societal perspective by including costs to patients of receiving treatment. Fourthly, following the release of GBD 2010 [26], new disability weights are available and the literature on drug effectiveness has since been updated. Finally, drugs such as simvastatin–which is listed on the national formulary and is currently available in Tanzania’s Medical Stores Department (MSD)–have come off patent, and so are more likely to be affordable and therefore policy relevant for Tanzania. In light of the above reasons, the purpose of the present study is to perform a cost-effectiveness analysis of the most relevant medical interventions for primary prevention of CVD in Tanzania using an absolute risk approach.

## Methods

### Model structure

Two Markov decision models for CVD risk without diabetes and CVD risk with diabetes–reflecting whether or not there is diabetes in addition to all other CVD risks–were constructed using TreeAge Pro 2014 software for four CVD risk cohorts. Further model details are specified in Appendix 1 to permit full replication of our results.

### Absolute risk of CVD

We constructed index cohorts representing each of the four CVD risk levels (see Appendix 1, Table 4) guided by the WHO’s prediction charts for AFRO E. By varying age, gender, blood pressure, cholesterol level, smoking and diabetes status we obtained hypothetical cohorts representing low, medium, high and very high CVD risk levels, respectively [22].

### Description of interventions

Table 1 below presents drug interventions included in the model.

**Table 1:** Drug interventions for primary prevention of CVD

Drug class	Acronym	Drug	Daily dosage
Angiotensin converting enzyme inhibitor	ACEI	Captopril	12.5 mg twice
Angiotensin receptor blocker	ARB	Losartan	50 mg once
Beta blocker	BB	Atenolol	50 mg once
Biguanide	Big	Metformin	500 mg thrice
Calcium channel blocker	CCB	Nifedipine	20 mg twice
Soluble aspirin	ASA	Aspirin	75 mg once
Statin	Sta	Simvastatin	40 mg once
Sulfonylureas	Sulf	Glibenclamide	5 mg once
Thiazide diuretics	Diu	Bendrofluazide	2.5 mg or 5 mg once

These drugs are recommended by the WHO’s CVD preventive guidelines [22], except for angiotensin receptor blocker (ARB), which we included because its patent recently expired. For an overview of the drug combinations considered, see Appendix 2.

### Input parameters

#### Transition probabilities

Annual risks for myocardial infarction (MI) and/or stroke were calculated from the Framingham Heart Study risk equations [27, 28] for the four different index cohorts. This was motivated by the absence of such data from the sub-Saharan region. Even though updated Framingham equations are available from the literature, these could not be applied in this work since the reported annual risks are combined for all CVD events [29, 30]. Age-specific background mortality rates were based on a Tanzanian life table [31] and were adjusted for the mortality attributable to CVD [32]. We used age and sex-specific case fatality rates from the WHO’s CHOosing Interventions that are Cost-Effective (CHOICE) study [33] (Appendix 3, Table 5).

Since stroke was classified according to severity, information about the probabilities of “first ever stroke” and “subsequent stroke” was necessary. These were derived from context-specific literature and if data was unavailable we sought expert opinions or made assumptions (Appendix 3, Table 6). We assumed that the probability of subsequent strokes after the second event was constant.

### Intervention costs

#### Cost of CVD prevention and treatment

The direct medical costs of providing medical primary prevention and cost to patients of receiving these services have been estimated elsewhere [34]. The provider cost of CVD treatment was identified and measured according to standard protocols from Arusha urban hospital using an ingredients approach. Resource valuation followed the opportunity cost method. Unit costs were estimated using activity-based and step-down methodologies. Patient cost of receiving CVD treatment was assumed to be 57.5 % higher [35] than the US$123 estimated as the patient cost of receiving CVD preventive measures [34]. The costing exercise follows a “narrow” societal perspective, whereby costs of health care, whether borne by the patient or the provider, are relevant. Since most economic evaluations are analyzed from the provider perspective only, a scenario analysis was conducted to explore the impact of this viewpoint on model recommendations. We assumed that primary preventive care can be performed within the existing facility setup and that upgrading of infrastructure is not required.

### Intervention effects

The intervention effects–in terms of relative risk (RR)–of drug classes used were retrieved from systematic reviews and meta-analyses of relevant randomized controlled trials, except for oral hypoglycemics which were based on one randomized controlled trial (RCT), see Appendix 3, Table 6 for references. Appendix 4 presents a summary of the comparators, RCT designs, statistical methods used and statements on primary prevention. The effects of interventions involving combinations of drugs were determined multiplicatively i.e. (RR_1_ × RR_2_ × RR_n_) [36]. It was assumed that intervention effects did not vary across the different underlying risk groups and that it was constant across all age groups. We also assumed perfect adherence to treatment.

### Health outcome

We used DALYs, which combine years lived with disability (YLDs) and years of life lost (YLLs), as our measure of health outcomes. YLDs were based on GBD 2010 disability weights of 0.422 for acute myocardial infarction and 0.021, 0.076 and 0.539 for mild, moderate and severe stroke respectively [26]. Tanzania’s sex-specific life expectancy in 2012 and a disability weight of 1 to reflect the dead health state were used to calculate YLLs.

See Table 2 below for a summary of parameters and sources.

**Table 2:** Model parameters and data sources

Parameter	Sources
Annual risk of MI or stroke - Table 5	Framingham Heart Study
Non-MI or non-stroke mortality rate - Table 5	Tanzanian 2012 life table and GHDx dataset
Fatality rate from MI and stroke - Table 5	WHO, CHOICE study
Cost of CVD prevention and treatment - Table 6	Authors’ previous study and primary analysis
Intervention costs and effects - Table 6	Tanzania MSD and meta-analyses
Disability weights - Table 6	Global burden of disease 2010
Other transition probabilities - Table 6	Cross-sectional studies and authors’ extrapolation

*MI* Myocardial infarction, *GHDx* Global Health Data Exchange, *WHO* World Health Organization, *CHOICE* CHOosing Interventions that are Cost-Effective, *MSD* Medical Stores Department

### Cost-effectiveness analysis

For the CVD risk both without and with diabetes, expected costs and outcomes were calculated for each of the possible interventions. Base-case results are presented as incremental costs and effects and incremental cost-effectiveness ratios (ICERs). Strategies having ICERs below US$610, which is Tanzania’s 2012 GDP per capita [37], (the lowest willingness to pay (WTP) value recommended by the WHO [38]) were considered “very cost-effective”. In our base case, health costs and outcomes were non-differentially discounted at 3 % annually. A scenario analysis was conducted to test whether and how results vary with differential discounting. Age weighting was not incorporated into the analysis due to criticisms raised in the GBD 2010 study [39].

### Sensitivity analyses

The rationale behind performing a sensitivity analysis is that the uncertainty of the model parameters is mainly due to lack of data or ambiguity regarding how the data were collected, simplifications and assumptions made [40, 41]. A set of one-way sensitivity analyses was performed to evaluate the impact of single assumptions about costs and outcomes on model results. Upper and lower variable ranges were taken from the reported 95 % confidence interval or calculated from the standard error when stated. Otherwise we assumed a range of +/− 15 % around the base case value. In the multivariate probabilistic uncertainty analysis, using a Monte Carlo simulation, we ran the model with distributions for each parameter rather than point estimates to determine the probability of optimal intervention being cost-effective against a range of cost-effectiveness thresholds [42, 43].

### Value of information analysis

High-quality evidence from multiple model input sources is not always available and decisions based on these model recommendations are subject to costly uncertainty. A value of information analysis allows for an estimation of the cost of existing uncertainty, which is determined by the function of the probability that a decision will be wrong for different levels of WTP for health and the size of the opportunity loss if the wrong decision is made [44]. We first calculated expected value of perfect information (EVPI) as the difference between the expected net benefit with perfect information and current information, and then we estimated population EVPI assuming an annual effective hypothetical population of 1000 patients, discounted at a rate of 3 % for 10 years.

## Results

### Base-case results

Table 3 below presents the base-case results for the two distinct models “CVD risk” and “CVD risk with diabetes” for the four CVD risk levels. In the table, all dominated and extendedly dominated strategies have been excluded. The complete tables presenting cost, incremental cost, effectiveness and incremental effectiveness of all strategies are available from the authors by request.

**Table 3:** Base case results for CVD risk without and with diabetes for four risk levels. All dominated strategies have been excluded

**CVD risk**											
Strategy	Cost	IC	Eff	IE	ICER	Strategy	Cost	IC	Eff	IE	ICER
**Low risk**						**Moderate risk**					
No treatment	461		0.00			No treatment	1516		0.00		
ACEI_Diu	1005	544	0.41	0.41	1327	ACEI_Diu	1683	167	1.02	1.02	164
ACEI_Diu_Sta	1259	254	0.49	0.08	3175	ACEI_Diu_Sta	1827	144	1.28	0.26	554
**High risk**						**Very high risk**					
No treatment	1695		0.00			No treatment	2028		0.00		
ACEI_CCB_Diu	2240	545	1.56	1.56	349	ACEI_CCB_Diu_ASA	3244	1216	2.44	2.44	498
ACEI_CCB_Diu_Sta	2404	164	1.83	0.27	607	ACEI_CCB_Diu_Sta_ASA	3433	189	2.73	0.29	652
**CVD risk with diabetes**											
Strategy	Cost	IC	Eff	IE	ICER	Strategy	Cost	IC	Eff	IE	ICER
**Low risk**						**Moderate risk**					
No treatment	964		0.00			No treatment	1805		0.00		
Sulf_ACEI_CCB	1481	517	0.85	0.85	608	Sulf_ACEI_CCB	1966	161	1.40	1.40	115
Big_Sulf_ACEI_CCB	1778	297	1.16	0.31	958	Big_Sulf_ACEI_CCB	2122	156	2.01	0.61	256
Big_Sulf_ACEI_CCB_Sta	2026	248	1.26	0.10	2480	Big_Sulf_ACEI_CCB_Sta	2311	189	2.21	0.20	945
**High risk**						**Very high risk**					
No treatment	1909		0.00			No treatment	2514		0.00		
Big_Sulf_ACEI_CCB	2576	667	2.16	2.16	309	Big_Sulf_ACEI_CCB_ASA	3696	1182	3.38	3.38	350
Big_Sulf_ACEI_CCB_Sta	2768	192	2.37	0.21	914	Big_Sulf_ACEI_CCB_Sta_ASA	3893	197	3.66	0.28	704
Big_Sulf_ACEI_ARB_CCB_Sta	3798	1030	2.47	0.10	10300	Big_Sulf_ACEI_ARB_CCB_Sta_ASA	4883	990	3.79	0.13	7615

*IC* Incremental cost, *Eff* Effectiveness, *IE* Incremental effectiveness, *ICER* Incremental cost-effectiveness ratio

For both models, simpler treatment combinations appear most cost-effective for lower CVD risks, while higher risks led to more complex drug combinations being cost-effective. Generally, managing CVD risk is more cost-effective for patients with higher risk than for patients with lower risk, and more cost-effective for patients with diabetes than for patients without diabetes (Table 3).

### CVD risk

#### Low risk

The results suggest that providing a combination of ACEI and Diu averts 0.41 DALYs at a cost of US$544 compared to no treatment, yielding the lowest ICER of US$1327 per DALY averted. Adding Sta to this duotherapy averted a further 0.08 DALYs for US$254.

#### Moderate risk

As for the low risk, ACEI and Diu yielded the lowest ICER of US$164 per DALY averted. Providing this intervention averts 1.02 DALYs at a cost of US$167 compared to giving no medical treatment.

#### High risk

The lowest ICER of US$349 per DALY averted was shown to result from providing a triple therapy of ACEI, CCB and Diu to high-risk patients compared to no treatment, whereby a cost of US$545 deterred 1.56 DALYs. Adding Sta to this combination requires an addition of US$607 per DALY averted.

#### Very high risk

Out of the three non-dominated strategies, a combination of ACEI, CCB, Diu and ASA yielded the lowest ICER value of US$498 per DALY averted compared to “no treatment”, by averting 2.44 DALYs for US$1216. Adding Sta to this combination required an additional US$189 to prevent a further 0.29 DALYs (Table 3).

### CVD risk with diabetes

#### Low risk

Model conclusions for this risk level suggest that a combination of Sulf, ACEI and CCB averts 0.85 DALYs at a lifetime cost of US$517, resulting in an ICER of US$608 per DALY averted. Adding Big to this combination will prevent a further 0.31 DALYs at an additional cost of US$297, with an ICER of US$958 per DALY averted.

#### Moderate risk

As for the low risk with diabetes, the triple therapy of one oral hypoglycemic and two anti-hypertensives yielded the lowest ICER of US$115 per DALY averted compared to not providing medical management. A more complex strategy of adding a second oral hypoglycemic required an additional US$156 to deter a further 0.61 DALYs with an ICER of US$256 per DALY averted.

#### High risk

Just over 2 DALYs are averted at a cost of US$667 by giving two oral hypoglycemics (Big & Sulf) and two anti-hypertensives (ACEI & CCB), with an ICER of US$309 per DALY averted. Additional DALYs are avoided at further cost and with more complex combinations. Adding Sta to this combination will further prevent 0.21 DALYs at a cost of US$192 while further addition of ARB deterred only 0.10 DALYs at a cost of US$1030. Corresponding ICERs are US$914 and US$10,300 per DALY averted respectively compared to the preceding interventions.

#### Very high risk

The lowest ICER was achieved by providing Big, Sulf, ACEI, CCB and ASA, whereby a cost of US$1182 deterred 3.38 DALYs with an ICER of US$350 per DALY averted.

### Representing uncertainty

In this sub-section, only the results for low and very high CVD risk without and with diabetes are presented, due to space limitations. For the results for moderate and high risks see Appendix 5.

### Deterministic sensitivity analysis

**Fig. 1:**
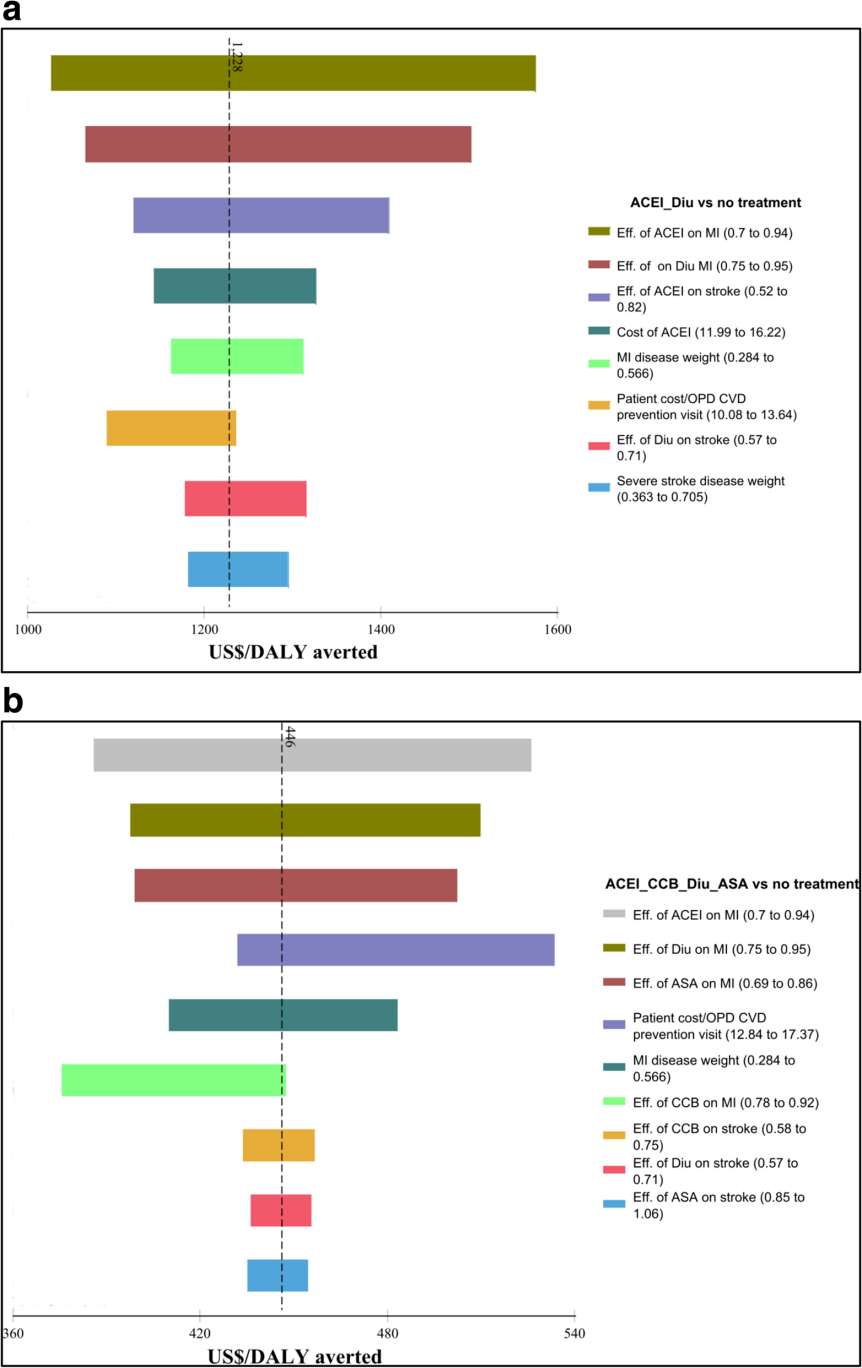
ICER tornado diagrams for low and very high CVD risk. **a** Low CVD risk. **b** Very high CVD risk

**Fig. 2:**
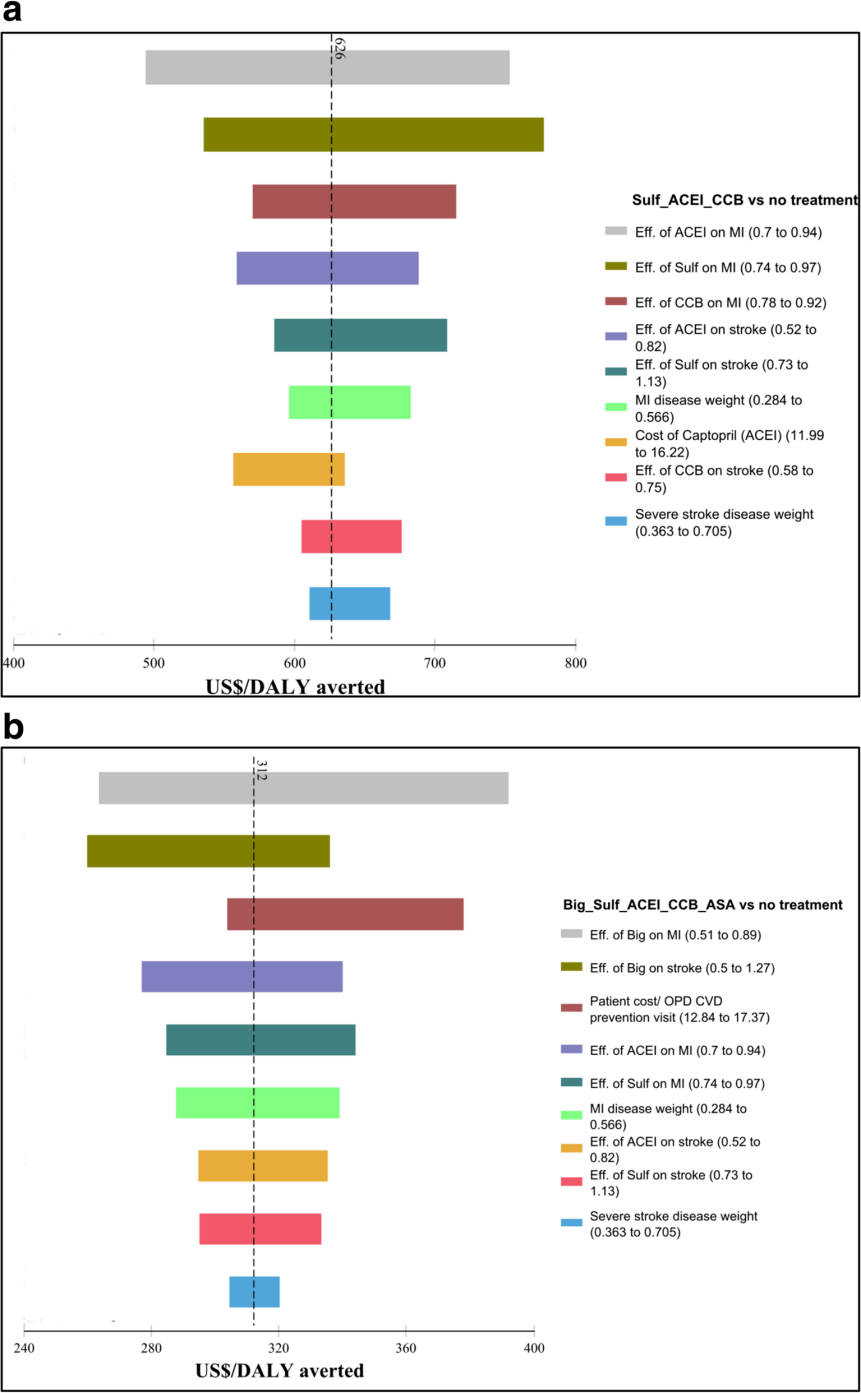
ICER tornado diagrams for low and very high CVD risk with diabetes. **a** Low CVD risk with diabetes. **b** Very high CVD risk with diabetes

Drug treatment effects are shown to be the most uncertain parameters for both low and very high CVD risk without and with diabetes. Variables representing less than 1 % of the total uncertainty were omitted since changing their assumptions had a negligible effect on the model. (Figs. 1 and 2).

### Probabilistic sensitivity analysis

**Fig. 3:**
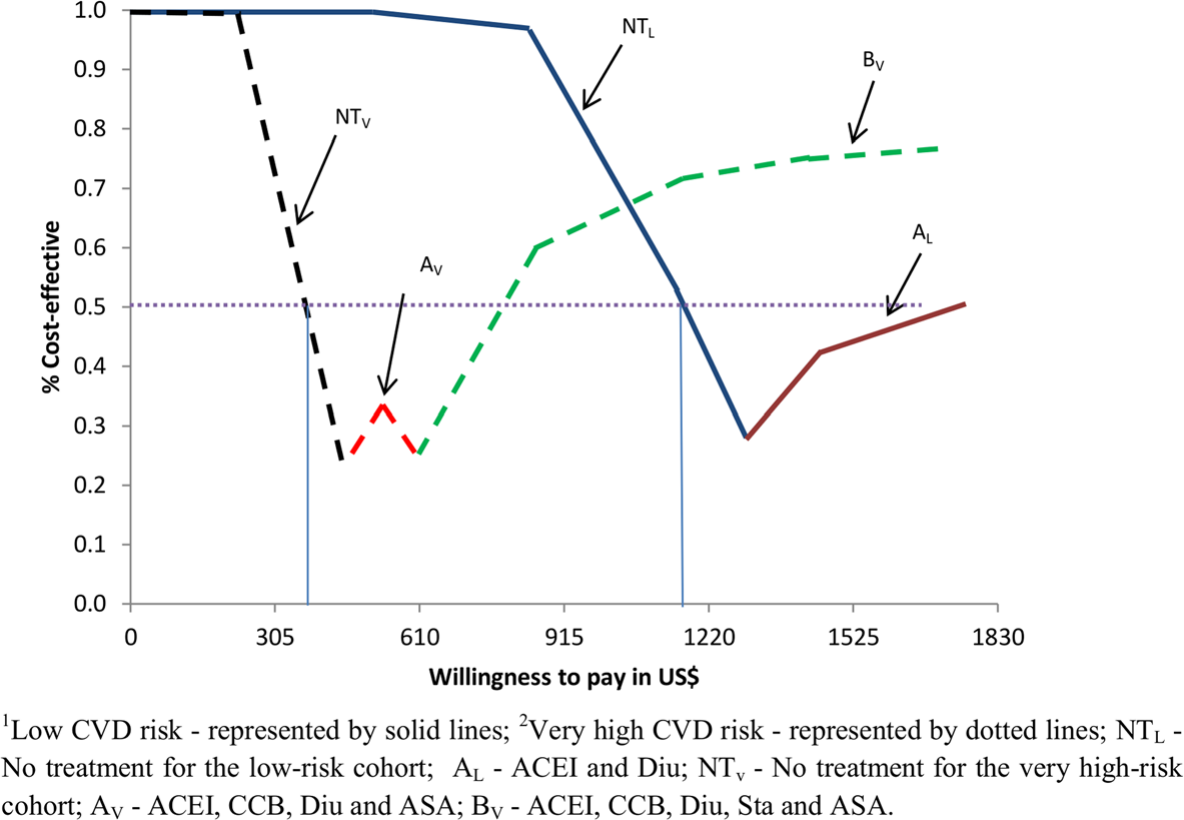
Cost-effectiveness acceptability frontier for low and very high CVD risk

**Fig. 4:**
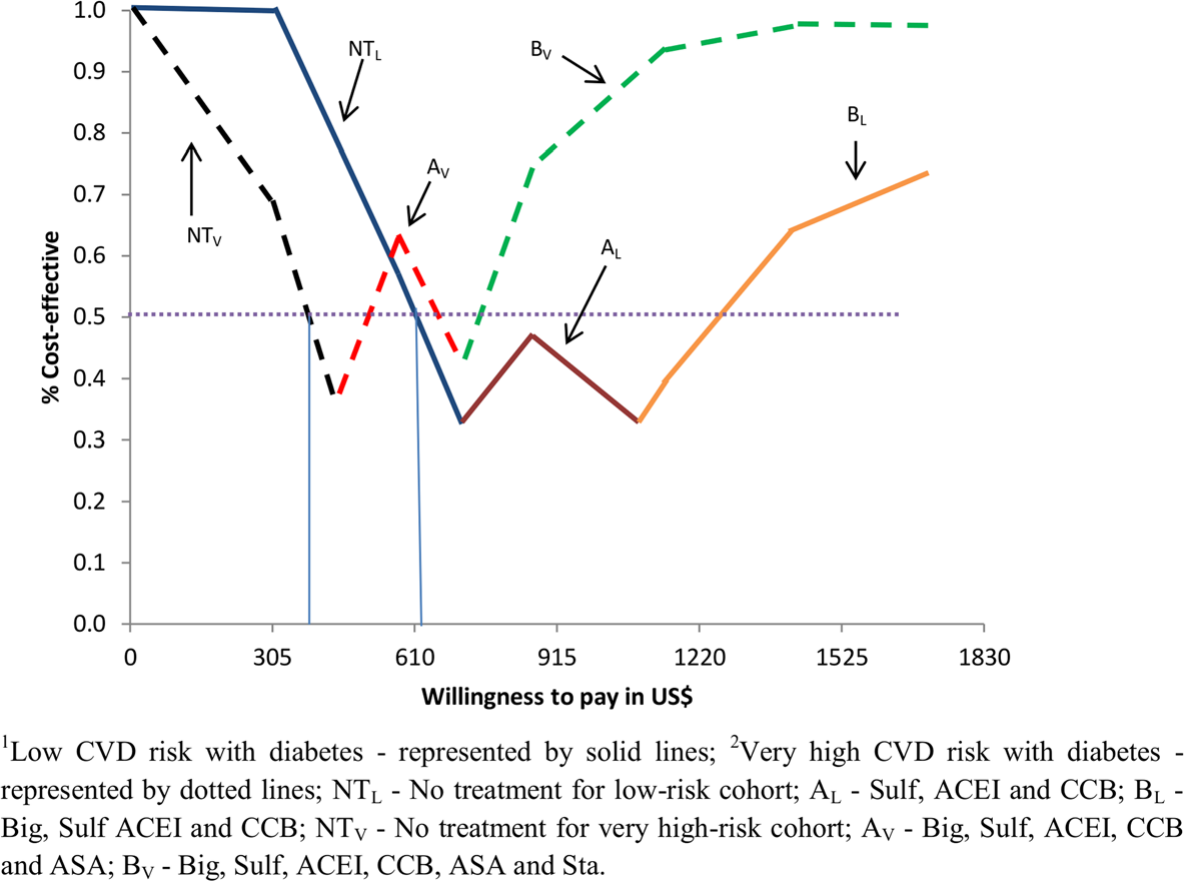
Cost-effectiveness acceptability frontier for low and very high CVD risk with diabetes

Figures 3 and 4 illustrate the optimal treatment paths, or cost-effectiveness acceptability frontiers (CEAFs), for low and very high CVD risk without and with diabetes for varying levels of plausible WTP to avert a DALY. For each level of WTP, the CEAFs illustrate only the optimal interventions with highest probability of being cost-effective, while all other alternatives are excluded.

CVD prevention is not likely to be cost-effective until WTP approaches US$1327 and US$498 per DALY averted in patients with low and very high CVD risk respectively. For low-risk patients, ACEI and Diu is optimal for all plausible levels of WTP higher than US$1327 per DALY averted, while for very high-risk patients a combination of ACEI, CCB, Diu and ASA is most likely to be optimal within the WTP range of US$498–US$651 per DALY averted, after which a further addition of Sta becomes optimal.

For patients with diabetes, WTP values of about US$608 and US$350 for low and very high CVD risk, respectively are required for CVD prevention to be cost-effective compared to CVD risk without diabetes. For low-risk patients, a combination of Sulf, ACEI and CCB is optimal for WTP values between US$608 and US$958, followed by Big, Sulf, ACEI and CCB for a ceiling ratio exceeding US$958. For very high-risk patients, a combination of Big, Sulf, ACEI, CCB and ASA is most likely to be optimal in the WTP range US$350–US$704 per DALY averted, after which the further addition of Sta becomes optimal.

The figures also illustrate that the above findings are surrounded by substantial uncertainty, except for patients with very high CVD risk and when WTP is very low (in which case no intervention is clearly optimal). For example, the ACEI and Diu combination in low-risk CVD patients without diabetes never exceeds a higher probability of being cost-effective than around 55 %, and consequently there is always at least a 45 % probability of the preferred strategy being sub-optimal. For low-risk patients with diabetes, the corresponding numbers are 75/25 % for the intervention Sulf, ACEI and CCB.

### Expected value of perfect information (EVPI) analysis

Presented here are the EVPI analysis results for low and very high CVD risk without and with diabetes. The population EVPI–which represents the maximum amount of funds a decision-maker, should be willing to invest in research to eliminate all uncertainties–varied considerably with the different values of the WTP and risk levels (Appendix 6).

**Fig. 5:**
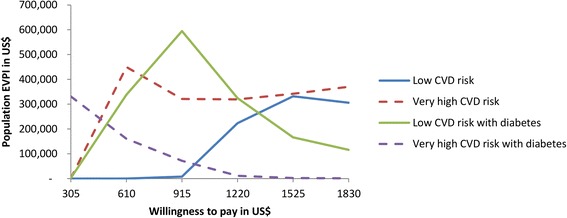
Population EVPI curve for low and very high CVD risk without and with diabetes

From Fig. 5 above, if Tanzanian’s WTP is US$610 then further research is potentially cost-effective for all risk levels except low CVD risk if the cost of proposed research is at most US$160,000.

### Scenario analyses

#### Societal vs provider perspective

##### CVD risk:

There were some changes in optimal therapies when the model was re-analyzed with patient costs excluded. For example, Diu as monotherapy became optimal for low and moderate risk levels, with ICERs of US$723 and US$092 per DALY averted respectively, and there was a re-emergence of BB in the non-dominated drug combinations for all risk levels (Appendix 7).

##### CVD risk with diabetes:

Model results remained largely the same for the CVD risk with diabetes model from the providers’ perspective. We observed the strategies Sulf, ACEI and CCB being recommended for high-risk patients and Sulf, ACEI, CCB and ASA for very high-risk patients, whereas such combinations were dominated in our base case (Appendix 7).

In both CVD risk without and with diabetes, model results from the societal perspective generally had lower ICERs compared to those from the provider viewpoint.

#### Differential vs non-differential discounting

Whether or not health outcomes are to be discounted (and at what rate) has raised a lot of debate in the field of economic evaluation [41, 45–48]. Our base-case analysis was performed using non-differential discounting of 3 % for costs and health outcomes. We applied differential discounting in two forms, a lower discount rate (1.5 %) and no discount rate for health outcomes, while keeping the discount rate for costs constant at 3 %. Lower discount rates for health outcomes reduce the ICERs, making CVD prevention more attractive, but model recommendations in terms of rank ordering of the alternatives did not change. With a lower or no discount rate for health outcomes, more costly yet more effective strategies become optimal for lower levels of WTP (Appendix 7).

#### Different index cohort definition

Our choice on index cohorts i.e. females for low risk and males for moderate to very high risk would imply that our main model results may not apply for males’ low-risk patients and females’ moderate to very high risks. We therefore constructed and re-run the model with a new set of index cohorts with the reverse gender and risk combinations (Appendix 8, Tables 11 and 12). The model conclusions remain robust in terms of rank ordering of the different drug combinations, but higher willingness to pay values are required in order to recommend interventions to female compared to male patients (Table 3 and Appendix 8, Table 13).

## Discussion

This analysis suggests that multi-drug combinations achieve the best value for money for almost all risk levels both in the CVD risk and CVD risk with diabetes cohorts. If Tanzania is willing to pay up to US$610–one Gross Domestic Product (GDP) per capita per DALY averted, below which interventions are considered to be very cost-effective by the WHO [38]–our model recommends no medical treatment for low-risk without diabetes patients. For a moderate risk level without diabetes, a combination of ACEI and Diu has an ICER that falls below the tentative WTP threshold value. Adding CCB to this combination was the optimal choice for high-risk patients, while for very high-risk patients further adding ASA is suggested. For patients with diabetes, a combination of Sulf, ACEI and CCB is predicted to be optimal for low and moderate risk levels while adding Big to this combination consistently yielded more health for money for high and very high risk levels. For very high-risk patients, ASA was also part of the combination.

These conclusions are in line with the WHO’s CVD preventive guidelines, which recommend no medical management for low CVD risks [22]. We also observed similar results to those demonstrated in the cost-effectiveness analysis of primary prevention of CVD in Australia, where they recommended Diu, CCB, ACEI and Sta for all patients with a 5 % 5-year CVD risk. The same conclusion cannot be drawn for our moderate-risk patients since we assumed that triple anti-hypertensives were not clinically relevant for this risk level.

Our results are different from those of a similar study performed in Tanzania [12], in which their recommended strategies, including combinations with BB, are dominated in our work. Differences in study perspectives, cost evidence, clinical evidence and choice of interventions analyzed meant that differences in optimal interventions were not unlikely. For example, interventions modelled in this work restricted ASA to very high-risk patients only and included ACEI in the drug combinations, which is not the case in the previous study. Comparing our model recommendations to the WHO-CHOICE study and a recent cost-effectiveness analysis to combat CVD in SSA and South East Asia results, we observed that drug combinations with Diu and BB were dominated and that our optimal strategies included combinations of ACEI with CCB or Diu. It is worth noticing that in these two studies only two anti-hypertensives, Diu and BB, were included, making comparability challenging [13, 49]. It suffices to say that we observed a re-emergence of BB in the recommended interventions in the scenario analysis from a provider perspective. This viewpoint was followed both in the former Tanzanian and WHO-CHOICE studies, making our results somewhat similar if the provider perspective was applied as base-case.

Our literature search revealed no studies analyzing options to prevent CVD in patients with diabetes. So we compared our results for this cohort with the WHO’s CVD guidelines, which recommend metformin (biguanide) as the oral hypoglycemic drug of choice. We find that glibenclamide (sulfonylureas) is optimal for low and moderate-risk patients, while adding metformin to glibenclamide is likely to be optimal for higher risk levels.

Our decision rule is based on the WHO’s proposed cost-effectiveness threshold of one times GDP per capita for Tanzania. This is because of the lack of a ceiling ratio set by Tanzania’s decision-makers. However, the use of WHO’s recommended cost-effectiveness benchmarks of one and three times GDP per capita [38] has received some criticisms. The basis of this threshold is unclear; as such, it is not known whether it infers values based on previous decisions or is set to optimally determine or exhaust the healthcare budgets of some representative countries [50]. Its use tends to make “almost” all healthcare interventions and programmes seem cost-effective and does not reflect the reality of resource constraints, hence challenging countries to introduce virtually any cost-effective interventions, which may end up with very low coverage and hence exacerbate healthcare inequalities [51]. Tanzania should preferably determine its own ceiling ratio by tailoring its recommendations to its own budgetary constraints, epidemiological and demographic profile, existing health infrastructure and other health-system considerations. Several approaches have been proposed to define the value of willingness to pay ceilings [50, 52, 53].

Most cost-effectiveness guidelines recommend a provider perspective in order to inform decisions about efficient use of healthcare resources [54]. The societal perspective has also been proposed in some guidelines because it is more consistent with welfare economics. Whether it is possible to fully adopt a societal perspective in cost-effectiveness analysis has been discussed [54–56]. We do not claim to have adopted a broad societal perspective, but a rather “narrow” one since we only expanded the providers’ perspective by including patient costs. The ICER results were more favourable from the societal than the provider perspective, which implies that ignoring patient costs could lead to sub-optimal resource allocation. It is equally true that including all relevant societal costs and benefits could significantly alter what we, in this work, consider to be very cost-effective.

Assuming the WTP value of US$610 and a proposed cost of research of about US$250,000, there will be value for further information for very high CVD risk without diabetes and low CVD risk with diabetes since their population EVPI exceeds the estimates of the proposed research. Current information regarding low CVD risk without diabetes is deem sufficient given its microscopic population EVPI value. Further exploration is required to determine what type of additional evidence would be most valuable and the most efficient research design [44]. In practice, though, the value of research will be lower since some uncertainty will always remain.

### Strengths and limitations

We adopt a “narrow” societal perspective in this study by taking into account patient costs. This viewpoint captures societal benefits that are not captured from more restricted viewpoints [56]. This work also analyzed interventions for all CVD risk levels, both without and with diabetes; although extensive, this permits comparisons within levels and between the two models for a better understanding of what does (or does not) work for each level of CVD risk.

These results should also be considered in light of model limitations. Firstly, the risk of myocardial infarction and stroke and drug treatment effects are based on data from trials carried out in developed countries, since good Tanzanian or regional data were unavailable. Secondly, effectiveness data for two drug classes (biguanide and glibenclamide) were extracted from one trial only, but again this was motivated by lack of data. Thirdly, we did not synthesize the evidence structure as we should have. Ideally, since most of our efficacy data were from trials with placebo as a comparator, indirect treatment comparisons (ITC) should have been performed and, in the very few instances where data was from other comparators, mixed treatment comparisons (MTC) should have been made. This weakness in inference concerning the relative efficacy of all treatments considered could possibly bias our results. However, these complex expansions of standard meta-analysis evidence are only as good as the trials included in them. Given the lack of a strong evidence base in our setting and since choices in healthcare resource allocation have to be made, we feel that our model results could, at this point, sufficiently inform these decisions [57]. Fourthly, we did not account for non-adherence to treatment in our model for reasons of simplification and lack of data, and our model may therefore not reflect real adherence in long-term treatment. Non-adherence would likely reduce the efficacy of the pharmacological drugs modelled, which would pull result in the direction of less favourable ICER estimates. On the other side, nonadherence represents cost savings, which would pull in the opposite direction. The overall effect on the estimates is an empirical question, but our intuition is that the former effect would weight more heavily, and that low adherence potentially would make ICERs somewhat less attractive. Lastly, we should expect the effectiveness values of our interventions to be lower in practice than the ones used in this work since the efficacy data are extracted from a “controlled” trial setting.

## Conclusion

Multiple drug combinations as a means of preventive cardiology yield ICERs lower than the proposed ceiling ratio of one GDP per capita for all risk levels except low CVD risk, which renders them very cost-effective. However, our model conclusions are surrounded by high uncertainty regarding both whether or not to treat and optimal treatment choice. The results of the value of information analysis suggest that it is potentially cost-effective to perform research to reduce uncertainties around model recommendations even at the lower WTP of US$610. Decision-makers could therefore simultaneously adopt the model recommendations and consider further research, since the adoption decision is reversible. The fact that interventions are cost-effective given current information does not automatically mean that they should be recommended for implementation, since health systems usually have other recourse competing “very cost-effective” interventions that have not been fully implemented. There are also other objectives besides maximizing population health outcomes. Budget impact analysis and distributional concerns should be considered, for example, to assess governments’ ability and to whom these benefits will accrue.

### Ethical statement

Ethical clearance was provided by the Ethical Review Committee of the Tanzania National Institute of Medical Research with Ref. No. NIMR/HQ/R.8 a/Vol. IX/1364. Respondents from the health facilities involved were asked for their consent to participate in the study and written permission was obtained prior to the interviews.

### Availability of data and materials

The datasets supporting the conclusions of this article are included within the article and its appendices.

## Figures and Tables

**Fig. 6:**
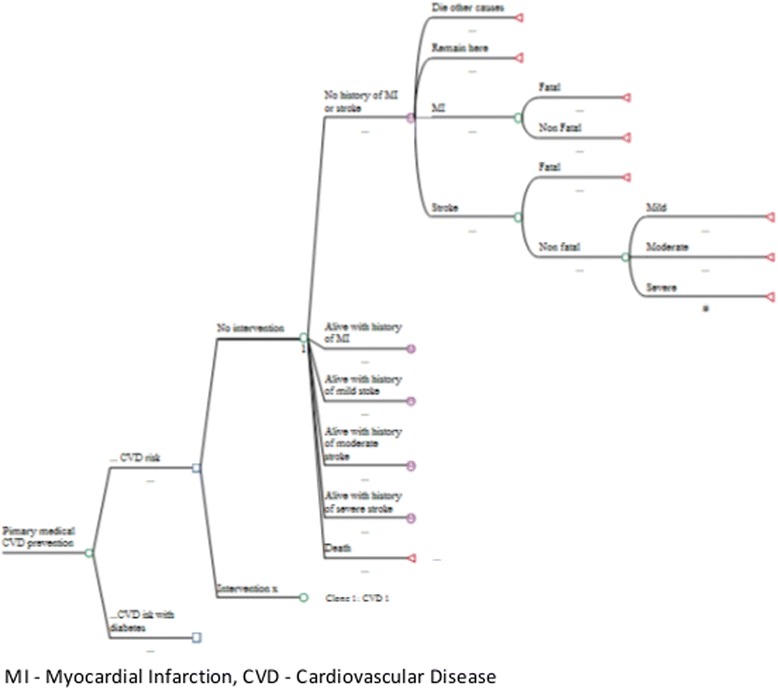
Model structure - CVD primary prevention

**Fig. 7:**
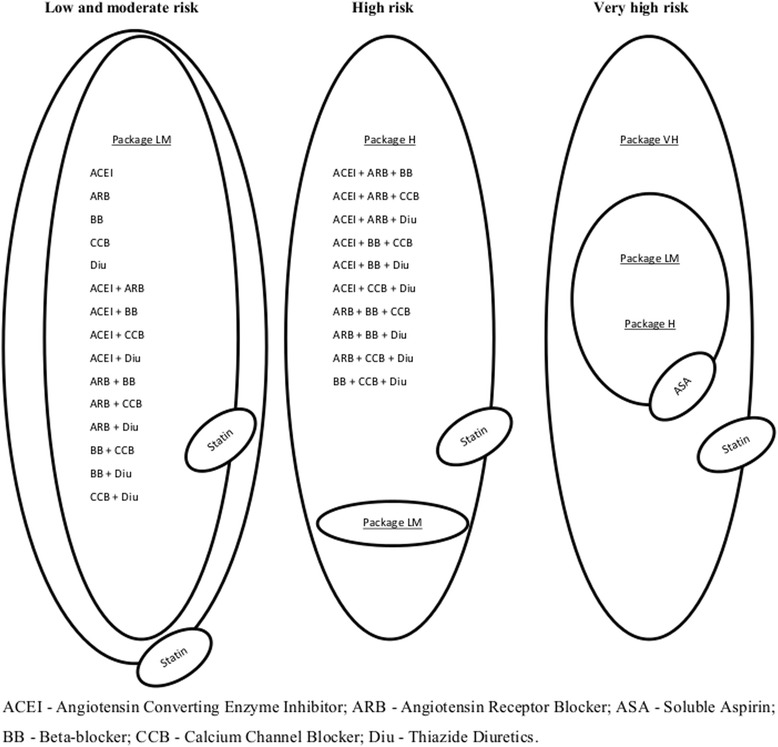
Interventions analysed for each CVD risk level

**Fig. 8:**
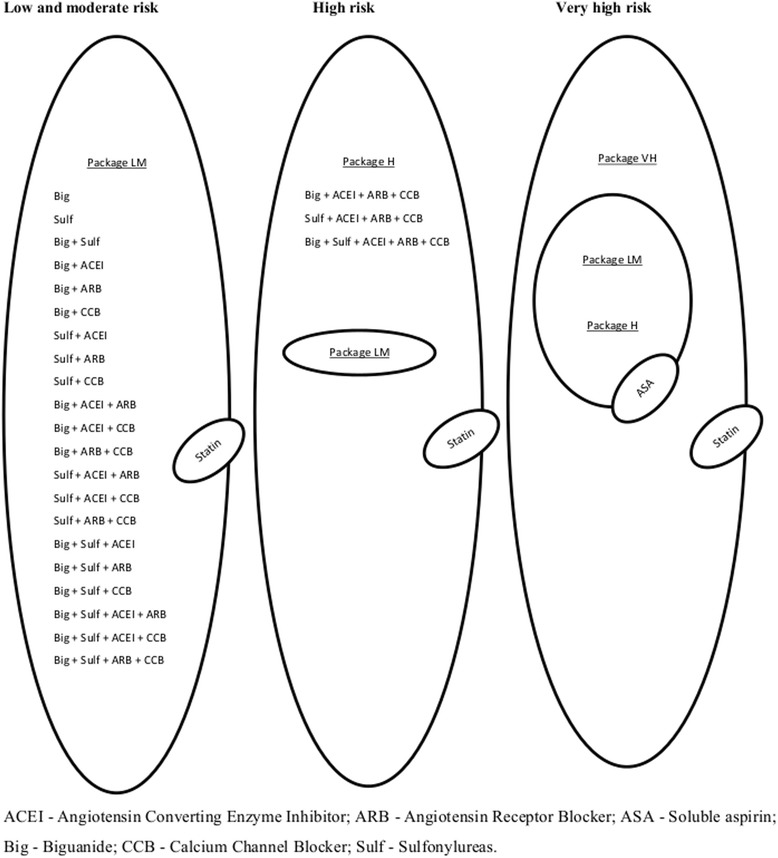
Interventions analysed for each CVD with diabetes risk level

**Fig. 9:**
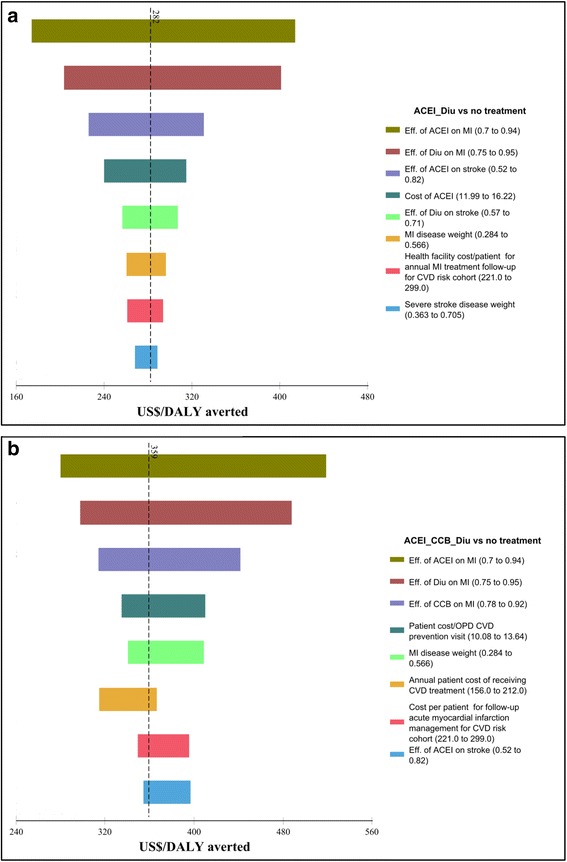
ICER tornado diagrams for moderate and high CVD risk. **a** Moderate CVD risk. **b** High CVD risk

**Fig. 10:**
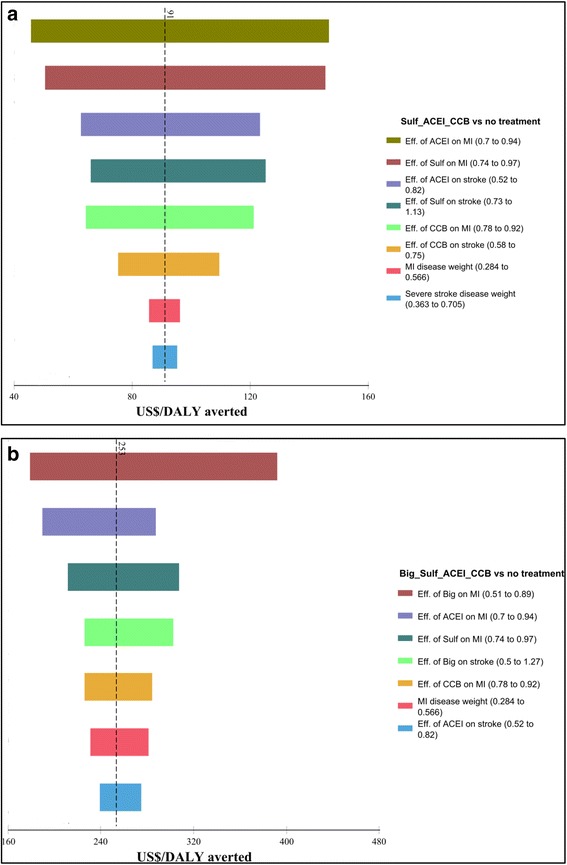
ICER tornado diagrams for moderate and high CVD risk with diabetes. **a** Moderate CVD risk with diabetes. **b** High CVD risk with diabetes

**Fig. 11:**
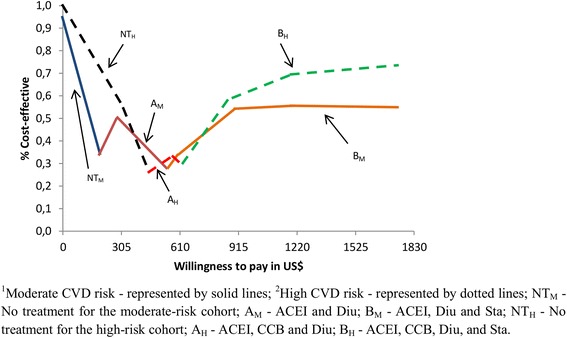
Cost effectiveness acceptability frontier for moderate and high CVD risk

**Fig. 12:**
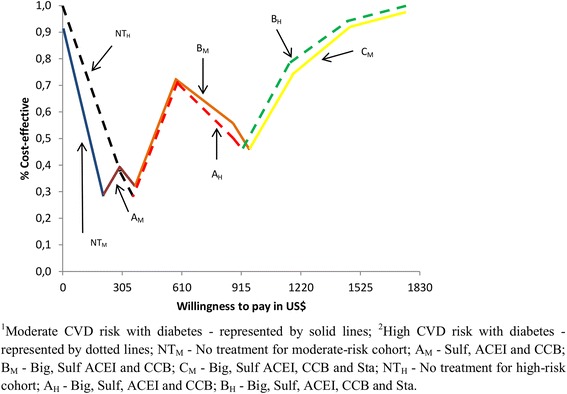
Cost effectiveness acceptability frontier for moderate and high CVD risk with diabetes

## Appendices

### Appendix 1

Cost-effectiveness model

The CVD prevention model is a probabilistic Markov model that provides information on health and healthcare costs over the lifetime of a hypothetical Tanzanian population (or population sub-groups). Two Markov decision models for CVD risk without diabetes and CVD risk with diabetes were constructed using TreeAge Pro 2014 software (Figure 6) for four CVD risk cohorts. Modelling them separately was necessary since CVD have different risk and treatment profiles depending on whether or not the patient is diabetic.

Six mutually exclusive health states were considered: “no history of CVD” (i.e. no previous myocardial infarction or stroke), “history of myocardial infarction”, and “history of stroke”. Finally, “death” was modelled as an absorbing state. Stroke was modelled in three different health states based on severity: “mild”, “moderate” or “severe”, in order to be consistent with disability weights reported in the GBD 2010 [26].

A cohort of healthy individuals with “no history of CVD” first enters the model at the age of 40 years. The cohort then transits between the different health states according to age-specific risks for each type of clinical event and depending on the risk reduction from the alternative preventive drugs. Each type of health state incurs costs and changes in health outcomes which are re-calculated after every annual cycle. A 60-year time horizon was considered to reflect the chronic nature of CVD.

In each model, “no treatment” was the baseline strategy and was compared with different medical interventions (singly or in combination) for primary prevention of CVD. The inclusion of “no treatment” as a comparator was important to allow for low-risk groups for whom treatment is not likely to be cost-effective. In order to explore the effects of the interventions in different populations, four distinct sub-models were created to represent four CVD risk levels: low risk, moderate risk, high risk and very high risk with a 10-year risk of CVD event of < 10 %, 10-19 %, 20-30 % and >30 % respectively.

### Appendix 2

Figure 7 presents the strategies analyzed in the CVD model diagrammatically. For low and moderate CVD risk, the strategies modelled are represented by “Package LM”, which consists of individual anti-hypertensives ACEI, ARB, BB, CCB and Diu in mono and duotherapy as shown by the inner circle in the diagram under the label “Low and moderate risk”. Package LM is then combined with statin, e.g. ACEI + statin, ACEI + ARB + statin etc. represented by the “statin circle” embedded in the inner circle. Third, statin alone is also analyzed for this risk level and lastly “no treatment” option is also included, making a total of 32 interventions modelled.

Interventions modelled for high risk consist of: (i) All the interventions under Package LM, i.e. the 15 anti-hypertensives in mono and duo therapy, (ii) “Package H” which is anti-hypertensives in triple therapy - a total of 10 options, (iii) Package LM and Package H combined with statin, e.g. ACEI + statin, ACEI + ARB + statin, ACEI + ARB + BB + statin. (iv) No treatment option. Fifty-one (51) interventions were analyzed for this risk level.

Lastly, strategies considered for very high-risk patients are interventions analyzed under Package LM and Package H but now combined with soluble aspirin, e.g. ACEI + ASA, ACEI + ARB + ASA, ACEI + ARB + BB + ASA, represented by the inner circle with “ASA circle” embedded within it in the diagram under “Very high risk”. These interventions are marked “Package VH”. All these combinations are then combined with statin, e.g. ACEI + ASA + statin, ACEI + ARB + ASA + statin, ACEI + ARB + BB + ASA + statin. With the no treatment option, the strategies made up a total of 51 interventions modelled.

The same approach can be used to interpret these diagrams, indicating the interventions analyzed for the four levels of CVD risk with diabetes. A total of 43 interventions were modelled for low and moderate CVD with diabetes risk levels while the number was 49 options for high and very high CVD with diabetes risk levels.

As depicted in Figure 8, drug combinations considered in the analysis consisted of up to two antihypertensives together with other drug classes for low and moderate risks. For these levels we assumed that combinations of three anti-hypertensives are clinically irrelevant. All other possible combinations without and with soluble aspirin were modelled for high and very high risks respectively. Soluble aspirin was included only for very high-risk patients, where it has been shown to be more beneficial than harmful [22].

### Appendix 3

**Table 5:** Annual risk of acute myocardial infarction and stroke, background mortality and case fatality rates

No previous history of acute myocardial infarction (AMI) or stroke								
CVD risk								
	Acute myocardial infarction				Stroke			
Age	Low	Moderate	High	Very high	Low	Moderate	High	Very high
40-49	0.0020	0.0130	0.0160	0.0230	0.0020	0.0050	0.0060	0.0100
50-59	0.0040	0.0190	0.0230	0.0310	0.0020	0.0050	0.0070	0.0110
60-69	0.0060	0.0290	0.0310	0.0400	0.0040	0.0070	0.0100	0.0150
70-79	0.0060	0.0360	0.0380	0.0440	0.0060	0.0110	0.0150	0.0220
80-89	0.0070	0.0390	0.0430	0.0520	0.0110	0.0170	0.0220	0.0330
90-99	0.0080	0.0440	0.0480	0.0570	0.0190	0.0190	0.0360	0.0460
CVD risk with diabetes								
40-49	0.0050	0.0140	0.0140	0.0290	0.0030	0.0060	0.0080	0.0130
50-59	0.0080	0.0210	0.0210	0.0380	0.0040	0.0070	0.0100	0.0150
60-69	0.0120	0.0270	0.0290	0.0440	0.0060	0.0100	0.0130	0.0200
70-79	0.0130	0.0290	0.0310	0.0460	0.0110	0.0150	0.0200	0.0290
80-89	0.0140	0.0350	0.0370	0.0570	0.0190	0.0220	0.0290	0.0420
90-99	0.0160	0.0390	0.0430	0.0620	0.0320	0.0290	0.0350	0.0630
With previous history of AMI or stroke								
CVD risk								
	Acute myocardial infarction				Stroke			
Age	Low	Moderate	High	Very high	Low	Moderate	High	Very high
40-49	0.0021	0.0143	0.0184	0.0276	0.0023	0.0059	0.0072	0.0133
50-59	0.0042	0.0209	0.0265	0.0372	0.0023	0.0059	0.0084	0.0146
60-69	0.0063	0.0319	0.0357	0.0480	0.0045	0.0082	0.0120	0.0200
70-79	0.0063	0.0396	0.0437	0.0528	0.0068	0.0129	0.0180	0.0293
80-89	0.0074	0.0429	0.0495	0.0624	0.0124	0.0199	0.0264	0.0439
90-99	0.0084	0.0484	0.0552	0.0684	0.0215	0.0222	0.0432	0.0612
CVD risk with diabetes								
40-49	0.0053	0.0154	0.0161	0.0348	0.0034	0.0070	0.0096	0.0173
50-59	0.0084	0.0231	0.0242	0.0456	0.0045	0.0082	0.0120	0.0200
60-69	0.0126	0.0297	0.0334	0.0528	0.0068	0.0117	0.0156	0.0266
70-79	0.0137	0.0319	0.0357	0.0552	0.0124	0.0176	0.0240	0.0386
80-89	0.0147	0.0385	0.0426	0.0684	0.0215	0.0257	0.0348	0.0559
90-99	0.0168	0.0429	0.0495	0.0744	0.0362	0.0339	0.0420	0.0838
Case Fatality rate								
	Acute myocardial infarction		Stroke		Background mortality			
Age	Males	Females	Males	Females	Age			
40-49	0.3040	0.4070	0.3470	0.3520	40-49	0.0112		
50-59	0.3110	0.4120	0.2480	0.1980	50-59	0.0120		
60-69	0.3360	0.4300	0.2830	0.2520	60-69	0.0225		
70-79	0.3670	0.4510	0.4200	0.4120	70-79	0.0467		
80-89	0.4090	0.4840	0.6420	0.6650	80-89	0.1006		
90-99	0.4510	0.5170	0.7920	0.8150	90-99	0.1458

**Table 6:** Intervention effects, costs, disability weights and other transition probabilities

Parameter	Model input§ *(95 % CI)*				Source
a. Intervention effects and costs					
Angiotensin converting enzyme inhibitor					
RR^1^	MI^2^	*0.81(0.70 - 0.94)*			[58]
	Stroke	*0.65(0.52 - 0.82)*			
Cost^3^		14.10			[59]
Angiotensin receptor blocker					
RR	MI	0.94 *(0.85 - 1.03)*			[60]
	Stroke	*0.91(0.85 - 0.98)*			
Cost		64.83			[59]
Beta-blocker					
RR	MI	0.90 *(0.78 - 1.03)*			[58]
	Stroke	0.83 *(0.72 - 0.97)*			
Cost		6.82			[59]
Biguanide					
RR	MI	0.67 *(0.51 - 0.89)*			[61]
	Stroke	0.80 *(0.50 - 1.27)*			
Cost		21.15			[59]
Calcium channel blocker					
RR	MI	0.85 *(0.78 - 0.92)*			[62]
	Stroke	0.66 *(0.58 - 0.75)*			
Cost		13.19			[59]
Soluble Aspirin					
RR	MI	0.77 *(0.69 - 0.86)*			[63]
	Stroke	0.95 *(0.85 - 1.06)*			
Cost		14.79			[59]
Statin					
RR	MI	0.86 *(0.82 - 0.90)*			[64]
	Stroke	0.90 *(0.85 - 0.95)*			
Cost		15.92			[59]
Sulfonylureas					
RR	MI	0.85 *(0.74 - 0.97)*			[61]
	Stroke	0.91 *(0.73 - 1.13)*			
Cost		6.37			[59]
Thiazide diuretics					
RR	MI	0.84 *(0.75 - 0.95)*			[58]
	Stroke	0.63 *(0.57 - 0.71)*			
Cost		1.18			[59]
b. Costs					
Cost of CVD^4^ prevention					
Patient cost per OPD^5^ visit		15.1			[34]¶
Provider cost per OPD visit		7.15			
Provider cost per lab test		4.07			
Cost of CVD treatment					
Patient cost per CVD treatment		194			[35]
	MI	Mild stroke	Mod.^6^ stroke	Sev.^7^ stroke	
CVD risk - first year	310	271	512	570	Estimation
CVD risk - subsequent years	260	67	76	313	
CVD risk with diabetes - first year	358	319	560	594	
CVD risk with diabetes - subsequent years	308	115	124	361	
c. Disability weights^8^					
Acute MI		0.42 *(0.28 - 0.57)*			[26]
Mild stroke		0.02 *(0.01 - 0.04)*			
Moderate stroke		0.08 *(0.05 - 0.11)*			
Severe stroke		0.54 *(0.36 - 0.71)*			
d. Other probabilities					
In the event of a first ever stroke,					
what is the probability of a:					
mild stroke for a CVD risk		0.19			[65, 66]
moderate for a CVD risk		0.44			Extrapolation
severe for a CVD risk		0.27			and
mild stroke for a CVD risk with diabetes		0.15			assumptions
moderate for a CVD risk with diabetes		0.35			
severe for a CVD risk with diabetes		0.50			
In the event of a subsequent stroke,					
what is the probability of:					
…mild stroke		0.15			
…moderate stroke		0.25			
…severe stroke		0.60			

^1^RR - Relative risk with beta distribution fitted; ^2^MI - Myocardial in arction; ^3^Cost - All costs are in 2012 US$ and were fitted with a gamma distribution; ^4^CVD - Cardiovascular disease; ^5^OPD - Out-patient department; ^6^Mod. - Moderate; ^7^Sev. - Severe; ^8^Beta distribution was fitted disability weights and other probabilities; §All model parameters were varied at ± 15 % unless stated; ¶ provider cost per OPD visit is the average cost per visit for the four facilities costed, provider cost per lab test available from Table 5, Patient cost per OPD visit was derived from annual patient cost divided by average number of clinic visits [34].

### Appendix 4

**Table 7:** Summary of meta-analyses and individual trials from which intervention effects were extracted

Drug class	Trials	Drug names	Comparator	Type	Blinded	Statistical method	Notes
ACEI	3	Ramipril, Deserpidine,	Placebo	MA	Double*	RR, M-H, fixed, Chi^2^	72 % PP
		Perindopril, Captopril					
ARB	17	Cardisartan, Valsartan,	Placebo	MA	NR~	RR, random, fixed, I^2^	NR
		Irbesartan, Telmisartan, Lorsartan					
ASA	6	Soluble aspirin	Placebo^1^	MA	Blindedƪ	Rate ratio, NS, *X* ^2^	PP trials only
BB	5	Propanolol, Atenolol	Placebo	MA	Double§	RR, M-H, fixed, Chi^2^	72 % PP
Big	1	Metformin	Conventional therapy	RCTs	No	NA	NA
CCB	22▪	NR	Placebo	MA	Double†	RR, M-H, fixed, Chi^2^	NR
Diu	19	Indapamide, CHTD, Chlorothiazide,	Placebo	MA	Double¶	RR, M-H, fixed, Chi^2^	72 % PP
		HCTZ, Bendrofluazide, T richloromethiazide					
Sta	10	Pravastatin, Lovastatin,	Placebo,UC^2^ or diet^3^	MA	Double‡	OR, random, fixed, I^2^	80 % PP or separate note on PP
		Artorvastatin, Simvastatin, Rosuvastatin					
Sulf	1	Sulfonylureas-insulin	Conventional therapy	RCTs	No	NA	NA

ACEI - Angiotensin Converting Enzyme Inhibitor, ARB - Angiotensin Receptor Blocker, Asp - Soluble Aspirin, BB - Beta Blocker, Big - Biguanide, CCB - Calcium Channel Blocker, Diu - Diuretic, Sta - Statins, Sulf - Sulfonylureas, CHTD - Chlorothalidone, HCTZ - Hydrochlorothiazide, UC - usual care, MA - Meta analyses, RCTs - Randomized Controlled Trials, NR - Not reported, RR - Relative risk, M-H - Mantel Haenszel, OR - Odds ratio, PP - Primary prevention, SP - Secondary prevention, NA - Not applicable, 22▪ - These are trials which reported MI events; 9 reported stroke events, Double* - two trials were open label, NR ~ − Even though blinding was not reported it was considered in trial quality, Placebo1 - Two trials compared aspirin with no comparator and open-label vitamin E, Blindedƪ - Two trials were open trials, Double§ - One trial was single blinded and one open trial, Double† - One trial was not blinded, Double¶ - Four trials were single blinded and three trials were open, Double‡ - Blinding status was not reported for one trial. For those drug classes which were not compared with placebo, we assume that comparability between different drug classes remains valid since a non- pharmacological agent was used. Ideally a mixed treatment comparison analysis should have been performed with all the drug classes whose comparator was not a placebo

### Appendix 6

**Table 8:** Expected Value of Perfect Information (EVPI) and population EVPI^1^ according to CVD risk levels

	Low risk		Moderate risk		High risk		Very high risk	
WTP value ($)	EVPI	pop EVPI	EVPI	pop EVPI	EVPI	pop EVPI	EVPI	pop EVPI
CVD risk								
305	0.0	0.0	44	382196	43	379456	0.2	1413
610	0.0	0.0	58	509069	62	545863	51	450067
915	01	7683	57	498410	47	412599	37	320778
1220	25	223152	67	590630	48	424051	36	319205
1525	38	331920	80	700692	52	457304	39	342096
1830	35	305884	93	820013	56	496261	42	370022
CVD risk with diabetes								
305	0.2	2027	55	482549	93	816305	38	330960
610	38	338058	14	123327	16	142351	18	160405
915	68	594776	21	182611	26	226298	08	71701
1220	37	324309	09	80525	07	63077	01	11007
1525	19	166059	03	22511	02	13393	0.2	2188
1830	13	115602	01	6711	0.3	2937	0.1	893

^1^ Population EVPI for 1000 patients, 10 years, 3 % discount rate; pop - population; WTP - Willingness to pay

### Appendix 7

**Table 9:** Provider perspective results for four CVD risk levels without and with diabetes. All dominated strategies have been excluded

**CVD risk**											
Strategy	Cost	IC	Eff	IE	ICER	Strategy	Cost	IC	Eff	IE	ICER
**Low risk**						**Moderate risk**					
No treatment	250		0.00			No treatment	855		0.00		
Diu	409	159	0.22	0.22	723	Diu	903	048	0.52	0.52	092
BB Diu	508	099	0.32	0.10	990	BB Diu	945	042	0.77	0.25	168
ACEI Diu	617	109	0.41	0.09	1211	ACEI Diu	1000	055	1.02	0.25	220
ACEI Diu Sta	884	267	0.49	0.08	3338	ACEI Diu Sta	1188	188	1.28	0.26	723
**High risk**						**Very high risk**					
No treatment	955		0.00			No treatment	1161		0.00		
ACEI BB Diu	1278	323	1.40	1.40	231	ACEI BB Diu ASA	1831	670	2.27	2.27	295
ACEI CCB Diu	1347	069	1.56	0.16	431	ACEI CCB Diu ASA	1905	074	2.47	0.20	370
ACEI CCB Diu Sta	1548	201	1.83	0.27	744	ACEI CCB Diu Sta ASA	2118	213	2.76	0.29	735
**CVD risk with diabetes**											
Strategy	Cost	IC	Eff	IE	ICER	Strategy	Cost	IC	Eff	IE	ICER
**Low risk**						**Moderate risk**					
No treatment	548		0.00			No treatment	1099		0.00		
Sulf ACEI CCB	1027	479	0.85	0.85	564	Sulf ACEI CCB	1304	205	1.40	1.40	146
Big Sulf ACEI CCB	1371	344	1.16	0.31	1110	Big Sulf ACEI CCB	1561	257	2.01	0.61	421
Big Sulf ACEI CCB Sta	1633	262	1.26	0.10	2620	Big Sulf ACEI CCB Sta	1783	222	2.21	0.20	1110
**High risk**						**Very high risk**					
No treatment	1160		0.00			No treatment	1516		0.00		
Sulf ACEI CCB	1479	319	1.52	1.52	210	Sulf ACEI CCB ASA	2067	551	2.54	2.54	217
Big Sulf ACEI CCB	1757	278	2.16	0.64	434	Big Sulf ACEI CCB	2365	298	3.36	0.82	363
Big Sulf ACEI CCB Sta	1978	221	2.37	0.21	1052	Big Sulf ACEI CCB Sta ASA	2584	219	3.64	0.28	782
Big Sulf ACEI ARB CCB Sta	3022	1044	2.47	0.10	10440	Big Sulf ACEI ARB CCB Sta ASA	3585	1001	3.77	0.13	7700

IC - Incremental cost; Eff - Effectiveness; IE - Incremental effectiveness; ICER - Incremental cost-effectiveness ratio

**Table 10:** Differential discounting results for CVD risk without and with diabetes for four risk levels. All dominated strategies have been excluded

	Base case	Lower DR	No DR		Base case	Lower DR	No DR
Strategy	oDR - 3 %	oDR - 1.5 %	oDR - 0 %	Strategy	oDR - 3 %	oDR - 1.5 %	oDR - 0 %
**CVD risk**							
**Low risk**	ICER			**Moderate risk**	ICER		
No treatment				No treatment			
ACEI_Diu	1327	907	585	ACEI_Diu	164	118	082
ACEI_Diu_Sta	3175	2121	1497	ACEI_Diu_Sta	554	379	262
**High risk**				**Very high risk**			
No treatment				No treatment			
ACEI_CCB_Diu	349	252	176	ACEI_CCB_Diu_ASA	498	364	258
ACEI_CCB_Diu_Sta	607	432	293	ACEI_CCB_Diu_Sta_ASA	652	461	315
**CVD risk with diabetes**							
**Low risk**				**Moderate risk**			
No treatment				No treatment			
Sulf_ACEI_CCB	608	465	311	Sulf_ACEI_CCB	115	083	058
Big_Sulf_ACEI_CCB	958	645	430	Big_Sulf_ACEI_CCB	256	179	123
Big_Sulf_ACEI_CCB_Sta	2480	1769	1126	Big_Sulf_ACEI_CCB_Sta	945	675	450
**High risk**				**Very high risk**			
No treatment				No treatment			
Big_Sulf_ACEI_CCB	309	222	154	Big_Sulf_ACEI_CCB_ASA	350	258	184
Big_Sulf_ACEI_CCB_Sta	914	640	436	Big Sulf ACEI CCB Sta ASA	704	505	346
Big Sulf ACEI ARB CCB Sta	10300	7357	4905	Big Sulf ACEI ARB CCB Sta ASA	7615	4950	3536

DR - Discount rate; ICER - Incremental cost-effectiveness ratio

### Appendix 8

**Table 11:** Index cohort’s characteristics according to CVD risk levels*

Index cohort	Low risk	Moderate risk	High risk	Very high risk
Without diabetes				
Total cholesterol	4 mmol	>8 mmol^1^	>8 mmol	>8 mmol
Systolic blood pressure	120-139 mmHg	140-159 mmHg^2^	160-179 mmHg	>180 mmHg
Smoking	No	No	No	Yes
Sex	Male	Female	Female	Female
With diabetes				
Total cholesterol	4 mmol	6 mmol	7 mmol^4^	>8 mmol
Systolic blood pressure	120-139 mmHg	140-159 mmHg^3^	160-179 mmHg	160-179 mmHg
Smoking	No	No	No	Yes
Sex	Male	Female	Female	Female

*These are the index characteristics of the genders not represented in tie main results. Age groups were: 40–49, 50–59, 60–69, 70–79, 80–89 and 90–99 (Table 12); ^1^7 mmol used for age group 40–49; ^2^160-179 mmHg applied to age group 40–49; ^3^160-179 mmHg used for age groups 40–59; ^4^6 mmol used for age groups > 60 years

**Table 12:** Annual risk of acute myocardial infarction and stroke for low CVD risk males and moderate to very high CVD risk females

No previous history of acute myocardial infarction (AMI) or stroke								
CVD risk								
	Acute myocardial infarction				Stroke			
Age	Low	Moderate	High	Very high	Low	Moderate	High	Very high
40-49	0.0040	0.0080	0.0100	0.0180	0.0040	0.0030	0.0030	0.0080
50-59	0.0060	0.0140	0.0160	0.0250	0.0040	0.0030	0.0040	0.0090
60-69	0.0100	0.0180	0.0210	0.0310	0.0050	0.0050	0.0060	0.0160
70-79	0.0140	0.0190	0.0230	0.0330	0.0080	0.0090	0.0110	0.0270
80-89	0.0190	0.0210	0.0250	0.0360	0.0130	0.0160	0.0190	0.0430
90-99	0.0230	0.0230	0.0270	0.0380	0.0150	0.0270	0.0320	0.0640
CVD risk with diabetes								
40-49	0.0060	0.0130	0.0160	0.0270	0.0050	0.0050	0.0050	0.0090
50-59	0.0090	0.0190	0.0230	0.0360	0.0050	0.0060	0.0060	0.0110
60-69	0.0140	0.0210	0.0250	0.0420	0.0070	0.0090	0.0110	0.0190
70-79	0.0190	0.0230	0.0270	0.0440	0.0110	0.0160	0.0190	0.0320
80-89	0.0240	0.0250	0.0290	0.0510	0.0170	0.0270	0.0320	0.0500
90-99	0.0290	0.0270	0.0310	0.0560	0.0190	0.0430	0.0500	0.0710
With previous history of AMI or stroke								
CVD risk								
	Acute myocardial infarction				Stroke			
Age	Low	Moderate	High	Very high	Low	Moderate	High	Very high
40-49	0.0042	0.0088	0.0115	0.0216	0.0045	0.0035	0.0036	0.0106
50-59	0.0063	0.0154	0.0184	0.0300	0.0045	0.0035	0.0048	0.0120
60-69	0.0105	0.0198	0.0242	0.0372	0.0057	0.0059	0.0072	0.0213
70-79	0.0147	0.0209	0.0265	0.0396	0.0090	0.0105	0.0132	0.0359
80-89	0.0200	0.0231	0.0288	0.0432	0.0147	0.0187	0.0228	0.0572
90-99	0.0242	0.0253	0.0311	0.0456	0.0170	0.0316	0.0384	0.0851
CVD risk with diabetes								
40-49	0.0063	0.0143	0.0184	0.0324	0.0057	0.0059	0.0060	0.0120
50-59	0.0095	0.0209	0.0265	0.0432	0.0057	0.0070	0.0072	0.0146
60-69	0.0147	0.0231	0.0288	0.0504	0.0079	0.0105	0.0132	0.0253
70-79	0.0200	0.0253	0.0311	0.0528	0.0124	0.0187	0.0228	0.0426
80-89	0.0252	0.0275	0.0334	0.0612	0.0192	0.0316	0.0384	0.0665
90-99	0.0305	0.0297	0.0357	0.0672	0.0215	0.0503	0.0600	0.0944

**Table 13:** Cost-effectiveness results for low CVD risk males and moderate to very high CVD risk females without and with diabetes. All dominated strategies have been excluded

**CVD risk**											
Strategy	Cost	IC	Eff	IE	ICER	Strategy	Cost	IC	Eff	IE	ICER
**Low risk**						**Moderate risk**					
No treatment	799		0.00			No treatment	991		0.00		
ACEI Diu	1202	403	0.61	0.61	661	ACEI Diu	1330	339	0.82	0.82	413
ACEI Diu Sta	1424	222	0.74	0.13	1708	ACEI Diu Sta	1525	195	1.02	0.20	975
**High risk**						**Very high risk**					
No treatment	1112		0.00			No treatment	1591		0.00		
ACEI CCB Diu	1941	829	1.22	1.22	680	ACEI CCB Diu ASA	3059	1468	2.30	2.30	638
ACEI CCB Diu Sta	2152	211	1.43	0.21	1005	ACEI CCB Diu Sta ASA	3277	218	2.56	0.26	838
**CVD risk with diabetes**											
Strategy	Cost	IC	Eff	IE	ICER	Strategy	Cost	IC	Eff	IE	ICER
**Low risk**						**Moderate risk**					
No treatment	1175		0.00			No treatment	1467		0.00		
Sulf ACEI CCB	1619	444	0.95	0.95	467	Sulf ACEI CCB	1756	289	1.36	1.36	213
Big Sulf ACEI CCB	1878	259	1.30	0.35	740	Big Sulf ACEI CCB	1971	215	1.94	0.58	371
Big Sulf ACEI CCB Sta	2110	232	1.42	0.12	1933	Big Sulf ACEI CCB Sta	2182	211	2.13	0.19	1111
**High risk**						**Very high risk**					
No treatment	1602		0.00			No treatment	2060		0.00		
Big Sulf ACEI CCB	2446	844	2.17	2.17	389	Big Sulf ACEI CCB ASA	3539	1479	3.37	3.37	439
Big Sulf ACEI CCB Sta	2654	208	2.38	0.21	991	Big Sulf ACEI CCB Sta ASA	3757	218	3.64	0.27	807
Big Sulf ACEI ARB CCB Sta	3700	1046	2.48	0.10	10460	Big Sulf ACEI ARB CCB Sta ASA	4770	1013	3.77	0.13	7792

IC - Incremental cost; Eff - Effectiveness; IE - Incremental effectiveness; ICER - Incremental cost-effectiveness ratio

## Abbreviations

ACEI: angiotensin converting enzyme inhibitor
AFRO E: African region E
ARB: angiotensin receptor blocker
ASA: soluble aspirin
BB: beta blocker
Big: biguanide
CCB: calcium channel blockers
CEAF: cost-effectiveness acceptability frontier
CHOICE: CHOosing interventions that are cost-effective
CVD: cardiovascular disease
DALY: disability- adjusted life years
Diu: diuretics
Eff: effectiveness
EVPI: expected value of perfect information
GBD: global burden of disease
GDP: gross domestic product
GHDx: Global Health Data Exchange
IC: incremental cost
ICER: incremental cost-effectiveness ratio
IE: incremental effectiveness
ITC: indirect treatment comparison
MI: myocardial infarction
MSD: Medical Stores Department
MTC: mixed treatment comparison
RCT: randomized control trial
RR: relative risk
SSA: sub-Saharan Africa
Sta: statin
Sulf: sulfonylureas
US$: United States of America dollar
WHO: World Health Organization
WTP: willingness to pay
YLDs: years lived with disability
YLLs: years of life lost
